# Risk and Outcome of Infective Endocarditis in Streptococcal Bloodstream Infections according to Streptococcal Species

**DOI:** 10.1128/spectrum.01049-23

**Published:** 2023-06-07

**Authors:** Hyeonji Seo, Junho Hyun, Haein Kim, Sunghee Park, Hyemin Chung, Seongman Bae, Jiwon Jung, Min Jae Kim, Sung-Han Kim, Sang-Oh Lee, Sang-Ho Choi, Yang Soo Kim, Yong Pil Chong

**Affiliations:** a Division of Infectious Diseases, Department of Internal Medicine, Hallym University Sacred Heart Hospital, Hallym University College of Medicine, Anyang, South Korea; b Division of Cardiology, Department of Internal Medicine, Asan Medical Center, University of Ulsan College of Medicine, Seoul, South Korea; c Department of Infectious Diseases, Asan Medical Center, University of Ulsan College of Medicine, Seoul, South Korea; University of Maryland School of Medicine

**Keywords:** bacteremia, echocardiography, infective endocarditis, *Streptococcus*, mortality

## Abstract

This study aimed to identify which streptococcal species are closely associated with infective endocarditis (IE) and to evaluate risk factors for mortality in patients with streptococcal IE. We performed a retrospective cohort study of all patients with streptococcal bloodstream infection (BSI) from January 2010 to June 2020 in a tertiary hospital in South Korea. We compared clinical and microbiological characteristics of streptococcal BSIs according to the diagnosis of IE. We performed multivariate analysis to evaluate the risk of IE according to streptococcal species and risk factors for mortality in streptococcal IE. A total of 2,737 patients were identified during the study period, and 174 (6.4%) were diagnosed with IE. The highest IE prevalence was in patients with Streptococcus mutans BSI (33% [9/27]) followed by S. sanguinis (31% [20/64]), S. gordonii (23% [5/22]), S. gallolyticus (16% [12/77]), and S. oralis (12% [14/115]). In multivariate analysis, previous IE, high-grade BSI, native valve disease, prosthetic valve, congenital heart disease, and community-onset BSI were independent risk factors for IE. After adjusting for these factors, S. sanguinis (adjusted OR [aOR], 7.75), S. mutans (aOR, 5.50), and S. gallolyticus (aOR, 2.57) were significantly associated with higher risk of IE, whereas S. pneumoniae (aOR, 0.23) and *S. constellatus* (aOR, 0.37) were associated with lower risk of IE. Age, hospital-acquired BSI, ischemic heart disease, and chronic kidney disease were independent risk factors for mortality in streptococcal IE. Our study points to significant differences in the prevalence of IE in streptococcal BSI according to species.

**IMPORTANCE** Our study of risk of infective endocarditis in patients with streptococcal bloodstream infection demonstrated that Streptococcus sanguinis, S. mutans, and S. gallolyticus were significantly associated with higher risk of infective endocarditis. However, when we evaluated the performance of echocardiography in patients with streptococcal bloodstream infection, patients with S. mutans and S. gordonii bloodstream infection had a tendency of low performance in echocardiography. There are significant differences in the prevalence of infective endocarditis in streptococcal bloodstream infection according to species. Therefore, performing echocardiography in streptococcal bloodstream infection with a high prevalence of, and significant association with, infective endocarditis is desirable.

## INTRODUCTION

Streptococcus species is one of the most common causes of infective endocarditis (IE) after Staphylococcus aureus (1, 2). Echocardiography has been the cornerstone of diagnosis of IE (3), and Sunnerhagen et al. (4) have developed a tool for identifying which patients with non-beta-hemolytic streptococcal bloodstream infections (BSIs) have a strong likelihood of developing IE to guide clinicians when to perform echocardiography. However, that study focused on non-beta-hemolytic streptococcal BSI and was based on the regional microbiology of Sweden. Deciding when to perform echocardiography to diagnose IE in streptococcal BSI is still challenging for clinicians.

Previous studies have investigated the frequencies of different streptococcal groups in streptococcal IE (5, 6). Although the introduction of matrix-assisted laser desorption ionization-time of flight mass spectrometry (MALDI-TOF) has made rapid and detailed identification of streptococcal species possible, only one study by Chamat-Hedemand et al. (7) has examined the prevalence of streptococcal IE according to streptococcal species.

In South Korea, Streptococcus species are the most common cause of IE followed by Staphylococcus aureus (8), and intravenous drug use-related IE is very rare because there are few intravenous drug users (8, 9).

Since the microbiologic etiologies of IE differ according to regions (1, 6, 7), we aimed to identify the risk of IE in patients with streptococcal BSI according to streptococcal species in an Asian country to assist with clinical decision-making in how far to pursue a diagnosis of IE. Furthermore, we sought to determine risk factors for mortality in patients with streptococcal IE.

## RESULTS

### Characteristics of patients with streptococcal BSI.

A total of 2,985 patients with streptococcal BSI were identified during the study period. Of these, 249 patients with streptococcal bacteremia due to unidentified species were excluded from the analysis, leaving 2,737 patients with streptococcal BSI included in the analysis. 10-year trends of streptococcal BSI according to species are presented in Fig. S1 in the supplemental material. The annual number of streptococcal BSI did not change significantly (*P = *0.243 for trend). Of the 2,737 patients, 1,177 (43%) underwent echocardiography within 1 month of streptococcal BSI, and 174 (6.4%; 95% confidence interval [CI], 5.5 to 7.3) were diagnosed with IE. As shown in Table S1, older patients (*P = *0.008) and those with high-grade BSI (*P < *0.001), a history of IE (*P < *0.001), and underlying heart disease were more likely to undergo echocardiography; those with community-onset BSI (*P < *0.001) and polymicrobial BSI (*P < *0.001) were less likely to undergo echocardiography. In terms of streptococcal species, patients with BSIs caused by S. mitis (*P < *0.001), S. oralis (*P < *0.001), S. gallolyticus (*P = *0.011), and S. sanguinis (*P = *0.015) were significantly more likely than other patients to undergo echocardiography, while those with BSIs with *S. anginosus* (*P = *0.009), S. pneumoniae (*P < *0.001), and *S. constellatus* (*P = *0.047) were significantly less likely.

### Prevalence of IE in streptococcal BSI, and risk factors for streptococcal IE.

**FIG 1:**
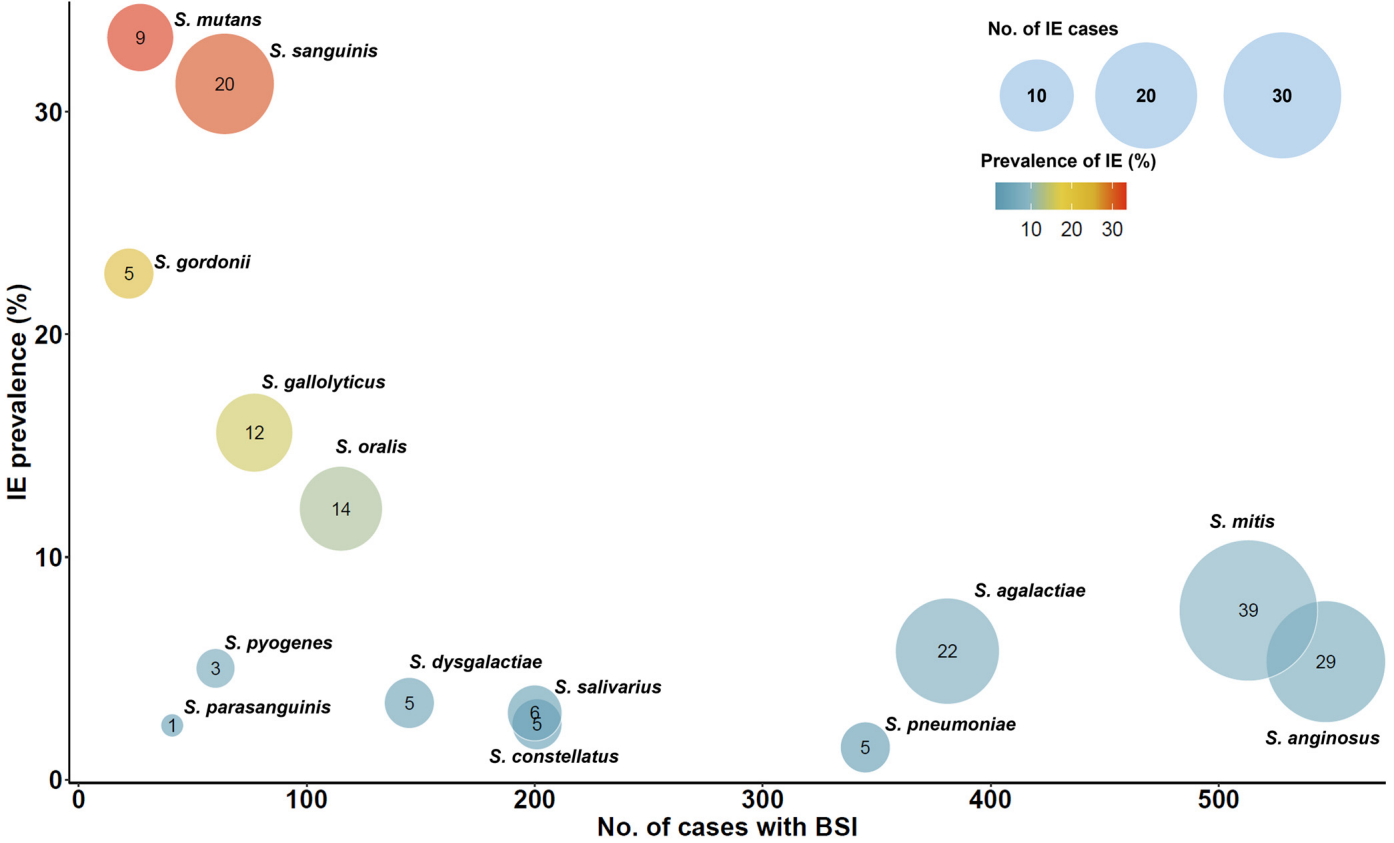
Prevalence of infective endocarditis in streptococcal bloodstream infections. BSI, bloodstream infections; IE, infective endocarditis; No, number. The figure shows the prevalence of infective endocarditis in streptococcal bloodstream infections according to streptococcal species. The horizontal axis represents numbers of bloodstream infections, and the vertical axis shows the prevalence of infective endocarditis. The size of each circle is proportional to the number of infective endocarditis cases caused by each streptococcal species, and the numbers inside each circle represents the actual number of infective endocarditis cases for that species. The color scale from blue to red corresponds to increasing prevalence of infective endocarditis.

Comparisons of the clinical and microbiological characteristics of the patients with IE and without IE are shown in Table 1. Patients with IE were more likely to have community-onset BSI (*P < *0.001), underlying heart disease (*P < *0.001), high-grade BSI (*P < *0.001), and history of IE (*P < *0.001). The highest IE prevalence was found in patients with Streptococcus mutans BSI (33% [9/27]) followed by S. sanguinis (31% [20/64]), S. gordonii (23% [5/22]), S. gallolyticus (16% [12/77]), and S. oralis (12% [14/115]) (Table 1 and Fig. 1). Mitis group streptococci were the most frequent viridans group streptococci causing IE, followed by the anginosus group, bovis group, and mutans group (Table 1). When beta-hemolytic streptococci were included, pyogenic group streptococci were the third largest, following the mitis group and anginosus group. Furthermore, when this analysis was restricted to those patients who underwent echocardiography, the overall results remained unchanged (Table S2).

**TABLE 1:** Comparison of patients with streptococcal bloodstream infections according to infective endocarditis (*n* = 2,737)^*a*^

Characteristic	Pt. with IE, *n* = 174 (%)	Pt. without IE, *n* = 2,563 (%)	*P* value
Age (yr), median (IQR)	58 (44–69)	61 (49, 70)	0.200
Male	97 (55.7)	1,461 (57.0)	0.746
Community-onset BSI	162 (93.1)	2,063 (80.5)	<0.001
Underlying disease			
Congenital heart disease	13 (7.5)	38 (1.5)	<0.001
Uncorrected	6 (3.4)	28 (1.1)	0.018
Corrected	7 (4.0)	10 (0.4)	<0.001
Native valve disease	41 (23.6)	98 (3.8)	<0.001
Prosthetic valve	39 (22.4)	30 (1.2)	<0.001
Cardiac device	4 (2.3)	14 (0.5)	0.024
Heart failure	19 (10.9)	98 (3.8)	<0.001
Ischemic heart disease	10 (5.7%)	89 (3.5)	0.119
High grade BSI	156 (89.7)	1,521 (59.3)	<0.001
Polymicrobial BSI	10 (5.7)^*b*^	595 (23.2)	<0.001
Previous history of IE	12 (6.9)	6 (0.2)	<0.001
Streptococcal group			
Anginosus group	34 (19.5)	745 (29.1)	0.007
Mitis group	79 (45.4)	668 (26.1)	<0.001
Pyogenic group	30 (17.2)	555 (21.7)	0.169
Salivarius group	6 (3.4)	198 (7.7)	0.038
Bovis group	12 (6.9)	75 (2.9)	0.004
Mutans group	9 (5.2)	18 (0.7)	<0.001
Other streptococci	5 (2.9)	340 (13.3)	<0.001
Streptococcal species			
*S. anginosus*	29 (16.7)	518 (20.2)	0.258
S. mitis	39 (22.4)	474 (18.5)	0.200
S. agalactiae	22 (12.6)	359 (14.0)	0.615
S. pneumoniae	5 (2.9)	340 (13.3)	<0.001
*S. constellatus*	5 (2.9)	196 (7.6)	0.019
S. dysgalactiae	5 (2.9)	140 (5.5)	0.140
*S. salivarius*	6 (3.4)	194 (7.6)	0.043
S. oralis	14 (8.0)	101 (3.9)	0.009
S. sanguinis	20 (11.5)	44 (1.7)	<0.001
*S. parasanguinis*	1 (0.6)	40 (1.6)	0.515
S. gallolyticus	12 (6.9)	65 (2.5)	0.003
S. pyogenes	3 (1.7)	57 (2.2)	>0.99
S. intermedius	0	41 (1.6)	0.109
S. mutans	9 (5.2)	18 (0.7)	<0.001
S. gordonii	5 (2.9)	17 (0.7)	0.011
Other	0	15 (0.6)	0.619
Valve surgery within 3 mo of BSI	93 (53.4)	27 (1.1)	<0.001
Recurrence of BSI within 6 mo	3 (1.7)	24 (0.9)	0.244
30-day mortality	10 (5.7)	323 (12.6)	0.007
90-day mortality	15 (8.6)	520 (20.3)	<0.001
1-yr mortality	40 (23.0)	1,160 (45.3)	<0.001

a Data are numbers of patients (with corresponding percentages shown in parentheses) unless otherwise specified. BSI, bloodstream infection; IE, infective endocarditis; IQR, interquartile range; Pt., patients.

b Of the 10 patients with polymicrobial BSI, 4 had coagulase-negative Staphylococcus bacteremia, 3 *Prevotella* spp, 1 Enterococcus faecalis, 1 Klebsiella pneumoniae, and 1 Acinetobacter baumannii bacteremia.

**FIG 2:**
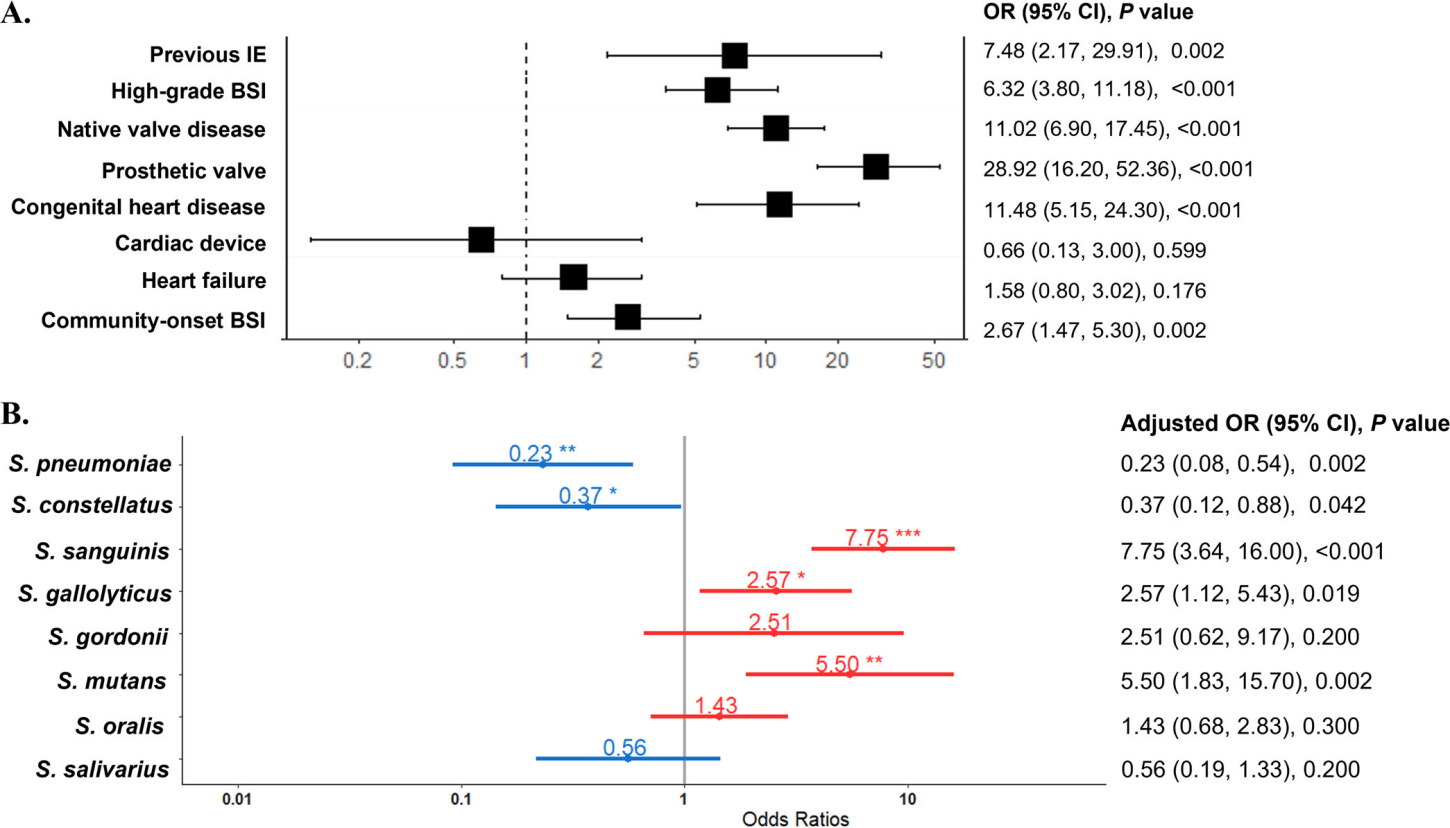
Risk of infective endocarditis in streptococcal bloodstream infections according to streptococcal species. BSI, bloodstream infections; CI, confidence interval; IE, infective endocarditis; OR, odds ratio. *, *P* value <0.05; **, *P* value <0.01; ***, *P* value <0.001. (A) Multivariate logistic regression analysis of the risk of infective endocarditis in streptococcal bloodstream infections. (B) The risk of infective endocarditis for different streptococcal species adjusted for the factors in panel A and for streptococcal species with significant univariate variables in Table 2. The results are given as adjusted odds ratio (95% confidence interval; *P* value).

In a multivariate analysis, previous IE, high-grade BSI, native valve disease, prosthetic valve, congenital heart disease, and community-onset BSI were independent risk factors for IE in streptococcal BSI (Fig. 2A). After adjusting for these risk factors, S. sanguinis (adjusted odds ratio [aOR], 7.75; 95% CI, 3.64 to 16.00; *P < *0.001), S. mutans (aOR, 5.50; 95% CI, 1.83 to 15.70; *P = *0.002), and S. gallolyticus (aOR, 2.57; 95% CI, 1.12 to 5.43; *P = *0.019) remained risk factors for IE, whereas S. pneumoniae (aOR, 0.23; 95% CI, 0.08 to 0.54; *P = *0.002) and *S. constellatus* (aOR, 0.37; 95% CI, 0.12 to 0.88; *P = *0.042) were associated with lower risk of IE (Fig. 2B).

### Outcome and risk factors for mortality in streptococcal IE.

One-year mortality due to streptococcal IE was 23% (40/174). Comparisons of the clinical and microbiological characteristics of deceased and surviving streptococcal IE patients are shown in Table 2. Patients who died were more likely than survivors to be older (*P < *0.001), have hospital-acquired BSI (*P = *0.021), a prosthetic valve (*P = *0.030), heart failure (*P = *0.003), ischemic heart disease (*P = *0.011), diabetes mellitus (*P = *0.006), a solid cancer (*P < *0.001), and chronic kidney disease (*P = *0.001). They were less likely to have congenital heart disease (*P = *0.041), undergo valve surgery within 3 months of BSI (*P < *0.001), or have a BSI with S. mitis (*P = *0.032) or mitis group bacteria (*P = *0.026).

**TABLE 2:** Characteristics of patients with infective endocarditis according to mortality (*n* = 174)^*a*^

Characteristic	Dead within 1 yr, *n* = 40 (%)	Survived, *n* = 134 (%)	*P* value
Age (yr), median (IQR)	70 (62–81)	55 (41–64)	<0.001
Male	17 (42.5)	80 (59.7)	0.055
Community-onset BSI	34 (85.0)	128 (95.5)	0.021
Underlying disease			
Congenital heart disease	0	13 (9.7)	0.041
Uncorrected	0	6 (4.5)	0.339
Corrected	0	7 (5.2)	0.354
Native valve disease	13 (32.5)	28 (20.9)	0.129
Prosthetic valve	14 (35.0)	25 (18.7)	0.030
Cardiac device	2 (5.0)	2 (1.5)	0.227
Heart failure	10 (25.0)	9 (6.7)	0.003
Ischemic heart disease	6 (15.0)	4 (3.0)	0.011
Diabetes mellitus	12 (30.0)	16 (11.9)	0.006
Solid cancer	12 (30.0)	9 (6.7)	<0.001
Liver cirrhosis	6 (15.0)	9 (6.7)	0.110
Chronic kidney disease	8 (20.0)	4 (3.0)	0.001
ESRD on renal replacement therapy	2 (5.0)	2 (1.5)	0.227
Chronic obstructive disease	2 (5.0)	2 (1.5)	0.227
Hematologic malignancy	2 (5.0)	0	0.052
Polymicrobial BSI	5 (12.5)	5 (3.7)	0.051
Previous history of IE	4 (10.0)	8 (6.0)	0.475
Streptococcal group			
Mitis group	12 (30.0)	67 (50.0)	0.026
Anginosus group	11 (27.5)	23 (17.2)	0.148
Pyogenic group	8 (20.0)	22 (16.4)	0.599
Bovis group	5 (12.5)	7 (5.2)	0.111
Salivarius group	2 (5.0)	4 (3.0)	0.622
Mutans group	1 (2.5)	8 (6.0)	0.686
Other streptococci	1 (2.5)	4 (3.0)	>0.99
Streptococcal species			
S. mitis	4 (10.0)	35 (26.1)	0.032
*S. anginosus*	9 (22.5)	20 (14.9)	0.259
S. agalactiae	6 (15.0)	16 (11.9)	0.609
S. sanguinis	2 (5.0)	18 (13.4)	0.169
S. oralis	4 (10.0)	10 (7.5)	0.740
S. gallolyticus	5 (12.5)	7 (5.2)	0.111
S. mutans	1 (2.5)	8 (6.0)	0.686
*S. salivarius*	2 (5.0)	4 (3.0)	0.622
*S. constellatus*	2 (5.0)	3 (2.2)	0.324
S. gordonii	2 (5.0)	3 (2.2)	0.324
S. pneumoniae	1 (2.5)	4 (3.0)	>0.99
*S. dysgalaciate*	1 (2.5)	4 (3.0)	>0.99
S. pyogenes	1 (2.5)	2 (1.5)	0.546
Valve surgery within 3 mo of BSI	11 (27.5)	82 (61.2)	<0.001
Recurrence of BSI within 6 mo	2 (5.0)	1 (0.7)	0.133

a Data are numbers of patients (with corresponding percentages shown in parentheses) unless otherwise specified. BSI, bloodstream infection; ESRD, end-stage renal disease; IE, infective endocarditis; IQR, interquartile range.

Because of the strong correlation between heart failure and ischemic heart diseases, we retained only ischemic heart disease in the multivariate analysis for mortality (Table 3). In addition, diabetes mellitus was highly correlated with chronic kidney disease. Thus, age, hospital-acquired BSI, prosthetic valve, ischemic heart disease, solid cancer, chronic kidney disease, valve surgery within 3 months of BSI, and nonmitis group were included in the Cox regression analysis. This indicated that age (adjusted hazard ratio [aHR], 1.04; 95% CI, 1.02 to 1.06; *P = *0.001), hospital-acquired BSI (aHR, 4.45; 95% CI, 1.80 to 11.02; *P = *0.001), ischemic heart disease (aHR, 4.15; 95% CI, 1.57 to 10.93; *P = *0.004), and chronic kidney disease (aHR, 2.83; 95% CI, 1.22 to 6.53; *P = *0.020) were independent risk factors for mortality in patients with streptococcal IE.

**TABLE 3:** Risk factors for mortality in patients with streptococcal infective endocarditis^*a*^

Risk factor	Univariate analysis		Multivariable analysis	
	HR (95% CI)	*P* value	Adjusted HR (95% CI)	*P* value
Age	1.06 (1.04–1.08)	<0.001	1.04 (1.02–1.06)	0.001
Hospital-acquired BSI	3.16 (1.32–7.53)	0.010	4.45 (1.80–11.02)	0.001
Prosthetic valve	2.11 (1.10–4.04)	0.024		
Ischemic heart disease	3.84 (1.61–9.18)	0.002	4.15 (1.57–10.93)	0.004
Solid cancer	3.67 (1.86–7.24)	<0.001	1.84 (0.81–4.16)	0.140
Chronic kidney disease	4.91 (2.24–10.80)	<0.001	2.83 (1.22–6.53)	0.020
Valve surgery within 3 mo of BSI	0.29 (0.14–0.58)	<0.001	0.57 (0.26–1.24)	0.160
Nonmitis group	2.10 (1.07–4.12)	0.032		

a BSI, bloodstream infection; CI, confidence interval; HR, hazard ratio.

Mortality rates in patients with streptococcal IE were significantly lower than in those with non-IE streptococcal BSI (Table 1 and Table S2). Patients with streptococcal IE were significantly less likely to have underlying diseases including solid cancer, diabetes mellitus, liver cirrhosis, chronic kidney disease, and hematologic malignancy than those with non-IE streptococcal BSI (Table S2).

## DISCUSSION

In the present study, infections with S. mutans had the highest prevalence of IE, followed by S. sanguinis, S. gordonii, S. gallolyticus, and S. oralis. This means that the probability of IE is high when these Streptococcus species are isolated in blood cultures, which is in agreement with the results of Chamat-Hedemand et al. (7) who observed a high prevalence of IE in BSIs with S. mutans, S. gordonii, S. sanguinis, S. gallolyticus, and S. oralis*/mitis*. Although they reported that patients with *S. parasanguinis* BSIs also had a high prevalence of IE (10.3%), the prevalence of *S. parasanguinis* in the present study was only 2.5%. In the present study, S. mutans, S. sanguinis, and S. gallolyticus were independent risk factors for IE in streptococcal BSI. However, when we compared patients with streptococcus BSI according to the use of echocardiography, patients with S. mutans or S. gordonii BSI were not more likely to undergo echocardiography than other patients. Based on these results, it seems desirable to perform routine echocardiography in streptococcal BSI caused by species with a high prevalence of, and significant association with, IE (e.g., S. mutans, S. sanguinis, S. gordonii, S. gallolyticus, and S. oralis).

In the present study, mitis group streptococci were the most frequent viridans pathogens in streptococcal IE, followed by the anginosus group, bovis group, and mutans group. This is partially consistent with previous studies that reported that the mitis group was the most frequent viridans group streptococci causing IE (5, 6). However, the anginosus group was previously reported to be less common among IE cases (9.0%). Whereas the mitis group was the most frequent streptococcal group causing IE in our study as well as in other studies (6, 17–19), the relatively large contribution of members of the anginosus group to streptococcal IE (20%) in our study is notable. Patients with streptococcus in the anginosus group were less likely to undergo echocardiography. Although the prevalence of IE among infections with streptococci in the anginosus group was low in our study, the absolute number of anginosus group patients with IE was the second largest due to the large proportion of the streptococcal BSI cases due to members of the anginosus group. Furthermore, the absolute number of pyogenic group patients with IE was the third largest. This was mainly attributed to S. agalactiae IE accounting for the third largest IE cases following S. mitis and *S. anginosus*. In a recent U.S. study of 296 patients with streptococcal IE, the pyogenic group accounted for 17% of streptococcal IE, and S. agalactiae was the most common pathogen (78%) in this pyogenic group (5). Therefore, it would be reasonable for clinicians to consider performing echocardiography in patients with infections caused not only by members of the mitis group, which led to a high prevalence and proportion of streptococcal IE, but also by those of the anginosus and pyogenic group, especially for patients with clinical risk factors for IE, such as community-onset, high-grade BSI, and underlying valve disease.

Although community-onset streptococcal BSI was independently associated with IE in the present study, such patients were significantly less likely to undergo echocardiography. An association of community acquisition with IE is not part of the Duke criteria (3). However, in a prospective cohort study, Murdoch et al. (1) reported that over 70% of cases of IE were community-acquired infections, and Sunnerhagen et al. (4) used community acquisition as one of the characteristics predictive of IE in non-beta-hemolytic streptococcal BSI. Our findings suggest that clinicians should be prepared to employ echocardiography in patients with community-acquired streptococcal BSI, especially when the patients have risk factors or other clinical characteristics favoring IE.

In multivariate analysis, age, hospital-acquired BSI, ischemic heart disease, and chronic kidney disease were independent risk factors for mortality in streptococcal IE. Although the clinical spectrum of IE has been evaluated in previous studies (20–22), the risk factors for mortality in streptococcal BSI have not been widely recognized. Suh et al. (23) reported that previous steroid use and immunosuppressant use were associated with increased mortality in viridans streptococcal IE. In a recent unpublished study, Chamat-Hedemand et al. (24) reported that renal disease at the time of IE diagnosis was significantly associated with 1-year mortality in streptococcal IE, which is in line with our findings. However, although they reported that bovis group infection was associated with lower mortality, we could not identify the association between particular streptococcal species and mortality due to the limited sample size. Recent valve surgery was associated with a higher chance of survival in univariate analysis, although it did not remain in multivariate analysis in our study. Previous studies reported the protective effect of surgery in patients with IE (1, 8, 25). However, these studies were not restricted to streptococcal IE and included patients with IE caused by S. aureus, enterococci, and others. Therefore, further large-scale studies investigating the role of surgery in streptococcal IE are needed.

It is noteworthy that mortality rates in patients with streptococcal IE were significantly lower than in those with non-IE streptococcal BSI. This may be because patients with streptococcal IE had significantly fewer comorbidities affecting mortality than those with non-IE streptococcal BSI.

Our study has several limitations. First, as it was a retrospective study, a diagnosis of IE might have been missed in patients who did not undergo echocardiography, and this might have affected the prevalence of IE according to species. However, since we reviewed the medical charts of every patient with streptococcal BSI, the possibility of false-negative diagnoses of IE is low. Second, we excluded patients with streptococcal BSI due to species that were not identified, which might have affected the prevalence of IE according to species, but since MALDI-TOF was introduced in our center in 2015, the proportion of patients with streptococcal BSI with unidentified species has been significantly reduced, and our results on the prevalence of IE are in line with those of a previous study (7). Furthermore, although the prevalence of IE was similar to that in the previous one (7), the results obtained by us reflect the etiologies of streptococcal BSI in Asia. Third, attributable mortality was not ascertained in our study, and the roles of surgical therapy and appropriate antimicrobial therapy have not been explored. Also, differences among Streptococcus species in antimicrobial susceptibility were not evaluated.

In conclusion, our findings suggest that there are significant differences in the prevalence of IE in streptococcal BSI according to species. Routine echocardiography in streptococcal BSI caused by S. mutans, S. sanguinis, S. gordonii, S. gallolyticus, and S. oralis could be beneficial due to the high prevalence of IE in these infections and their association with IE. Furthermore, echocardiography might be considered in patients with streptococcal BSI caused by the mitis, anginosus and pyogenic groups who have clinical risk factors for IE.

## MATERIALS AND METHODS

### Study population and design.

We retrospectively collected the data of all the patients with streptococcal BSI at Asan Medical Center, a 2,700-bed tertiary referral hospital in Seoul, South Korea, from January 2010 to June 2020. In patients suspected of having BSI, it was recommended to collect at least three sets of blood cultures from different venipuncture sites before administration of antibiotics. In patients where IE was suspected based on the modified Duke criteria, echocardiography, primary transthoracic, was performed. For patients with a history of valvular heart diseases or those exhibiting negative findings on transthoracic echocardiography, transesophageal echocardiography was employed. Patients with suspected IE were managed by a multidisciplinary team including cardiologists, cardiovascular surgeons, and infectious diseases specialists. Antibiotics and surgical intervention were decided according to the American Heart Association and the European Society of Cardiology guidelines (10, 11).

Only the first episode of BSI caused by streptococcal species was included in the analysis. Patients were excluded if the causative streptococci were not identified to the species level. Clinical data were collected from electronic medical records and included the following: demographics, echocardiographic results within 1 month of BSI, preexisting medical conditions, microbiological data, valve surgery within 3 months of BSI, and outcomes. We compared the clinical and microbiological characteristics of patients who underwent echocardiography within 1 month of streptococcal BSI and those who did not, followed by the clinical and microbiological characteristics of patients according to diagnosis of IE.

### Patient consent.

This observational study was approved by the Institutional Review Board of Asan Medical Center (IRB no. 2022-1244). Informed consent was waived by the ethics committee of Asan Medical Center because no intervention was involved, and no patient-identifying information was included. To protect personal privacy, identifying information in the electronic database was encrypted.

### Study definitions and outcome.

Streptococcal BSI was defined as isolation of Streptococcus from one or more blood cultures. Community-onset BSI was classified as blood culture obtained within 48 h of hospitalization (12). High-grade BSI was defined as positive results obtained in more than half of the blood culture bottles, with at least two bottles giving positive results (13). Polymicrobial BSI was defined as isolation of organisms other than Streptococcus during the streptococcal BSI. Patients with infective endocarditis (IE) were diagnosed based on clinical data and echocardiographic results according to the modified Duke criteria, and cases with Duke-possible IE who had no evidence of vegetation on echocardiography were not classified as IE (14). Native valve diseases included aortic stenosis, aortic insufficiency, mitral stenosis, and mitral insufficiency (7). Patients suffering from both congenital heart disease and native valve disease were classified as congenital heart disease and were excluded from native valve disease. Patients with both congenital heart disease and prosthetic valve were classified as congenital heart disease and excluded from prosthetic valve. Patients with chronic kidney disease included those with end-stage renal disease on renal replacement therapy. The primary outcome was 1-year crude mortality.

### Microbiological data.

All blood culture samples were processed by the hospital microbiology laboratory using a standard blood culture system (Bactec 9240 or Bactec FX; Becton, Dickinson, NJ, USA). Species and antimicrobial susceptibilities were determined with a Vitek (bioMérieux, France) or Microscan (Beckman Coulter, CA, USA) in accordance with the standard criteria of the Clinical and Laboratory Standards Institute (15). MALDI-TOF (MS, Bruker, Bremen, Germany) was implemented for species identification from 2015 in our center. Streptococcal species were divided into five groups based on their phylogenetic relationships (16). The anginosus group included *S. anginosus*, *S. constellatus*, and S. intermedius; the mitis group included S. mitis, S. sanguinis, S. oralis, *S. parasanguinis*, S. gordonii, and *S. cristatus*; the pyogenic group included S. agalactiae, S. dysgalactiae, and S. pyogenes; the salivarius group included *S. salivarius*, and *S. vestibularis*; the bovis group included *S. alactolyticus*, S. equinus, S. gallolyticus, and *S. lutentiensis*; and the mutans group included only S. mutans.

### Statistical analysis.

Trends in frequency of streptococcal BSI according to streptococcal species were evaluated with the Mann-Kendall trend test. Prevalences of IE at the streptococcal species level were calculated as number of IE cases due to each species divided by total number of BSI caused by that streptococcal species during the study period (7). Student's *t* test or the Mann-Whitney *U* test was used to compare continuous variables, and the Pearson chi-squared test or Fisher’s exact test was used to compare the respective categorical ones, as appropriate. We performed multivariate analysis to identify risk factors for IE in patients with streptococcal BSI using logistic regression. To evaluate the risk factors for mortality in streptococcal IE, we performed a Cox regression analysis. A two-tailed *P* value of <0.05 was considered statistically significant. All statistical analyses were performed with R software, version 3.3.3 (R Development Core Team, Vienna, Austria).

## References

[1] Murdoch DR, Corey GR, Hoen B, Miró JM, Fowler VG, Jr, Bayer AS, Karchmer AW, Olaison L, Pappas PA, Moreillon P, Chambers ST, Chu VH, Falcó V, Holland DJ, Jones P, Klein JL, Raymond NJ, Read KM, Tripodi MF, Utili R, Wang A, Woods CW, Cabell CH, International Collaboration on Endocarditis-Prospective Cohort Study (ICE-PCS) Investigators. 2009. Clinical presentation, etiology, and outcome of infective endocarditis in the 21st century: the International Collaboration on Endocarditis-Prospective Cohort Study. Arch Intern Med 169:463–473. doi:10.1001/archinternmed.2008.603. 19273776 PMC3625651

[2] Olmos C, Vilacosta I, Fernández-Pérez C, Bernal JL, Ferrera C, García-Arribas D, Pérez-García CN, San Román JA, Maroto L, Macaya C, Elola FJ. 2017. The evolving nature of infective endocarditis in Spain: a population-based study (2003 to 2014). J Am Coll Cardiol 70:2795–2804. doi:10.1016/j.jacc.2017.10.005. 29191329

[3] Li JS, Sexton DJ, Mick N, Nettles R, Fowler VG, Jr, Ryan T, Bashore T, Corey GR. 2000. Proposed modifications to the Duke criteria for the diagnosis of infective endocarditis. Clin Infect Dis 30:633–638. doi:10.1086/313753. 10770721

[4] Sunnerhagen T, Törnell A, Vikbrant M, Nilson B, Rasmussen M. 2018. HANDOC: a handy score to determine the need for echocardiography in non-β-hemolytic streptococcal bacteremia. Clin Infect Dis 66:693–698. doi:10.1093/cid/cix880. 29040411

[5] Kim SL, Gordon SM, Shrestha NK. 2018. Distribution of streptococcal groups causing infective endocarditis: a descriptive study. Diagn Microbiol Infect Dis 91:269–272. doi:10.1016/j.diagmicrobio.2018.02.015. 29567126

[6] Nilson B, Olaison L, Rasmussen M. 2016. Clinical presentation of infective endocarditis caused by different groups of non-beta haemolytic streptococci. Eur J Clin Microbiol Infect Dis 35:215–218. doi:10.1007/s10096-015-2532-5. 26610338

[7] Chamat-Hedemand S, Dahl A, Østergaard L, Arpi M, Fosbøl E, Boel J, Oestergaard LB, Lauridsen TK, Gislason G, Torp-Pedersen C, Bruun NE. 2020. Prevalence of infective endocarditis in streptococcal bloodstream infections is dependent on streptococcal species. Circulation 142:720–730. doi:10.1161/CIRCULATIONAHA.120.046723. 32580572

[8] Kim JH, Lee HJ, Ku NS, Lee SH, Lee S, Choi JY, Yeom JS. 2021. Infective endocarditis at a tertiary care hospital in South Korea. Heart 107:135–141. doi:10.1136/heartjnl-2020-317265. 33033067 PMC7788257

[9] Lee MR, Chang SA, Choi SH, Lee GY, Kim EK, Peck KR, Park SW. 2014. Clinical features of right-sided infective endocarditis occurring in non-drug users. J Korean Med Sci 29:776–781. doi:10.3346/jkms.2014.29.6.776. 24932077 PMC4055809

[10] Baddour LM, Wilson WR, Bayer AS, Fowler VG, Jr, Tleyjeh IM, Rybak MJ, Barsic B, Lockhart PB, Gewitz MH, Levison ME, Bolger AF, Steckelberg JM, Baltimore RS, Fink AM, O'Gara P, Taubert KA, American Heart Association Committee on Rheumatic Fever, Endocarditis, and Kawasaki Disease of the Council on Cardiovascular Disease in the Young, Council on Clinical Cardiology, Council on Cardiovascular Surgery and Anesthesia, and Stroke Council. 2015. Infective endocarditis in adults: diagnosis, antimicrobial therapy, and management of complications: a scientific statement for healthcare professionals from the American Heart Association. Circulation 132:1435–1486. doi:10.1161/CIR.0000000000000296. 26373316

[11] Habib G, Lancellotti P, Antunes MJ, Bongiorni MG, Casalta JP, Del Zotti F, Dulgheru R, El Khoury G, Erba PA, Iung B, Miro JM, Mulder BJ, Plonska-Gosciniak E, Price S, Roos-Hesselink J, Snygg-Martin U, Thuny F, Tornos Mas P, Vilacosta I, Zamorano JL, ESC Scientific Document Group. 2015. 2015 ESC Guidelines for the management of infective endocarditis: the Task Force for the Management of Infective Endocarditis of the European Society of Cardiology (ESC). Endorsed by: European Association for Cardio-Thoracic Surgery (EACTS), the European Association of Nuclear Medicine (EANM). Eur Heart J 36:3075–3128. doi:10.1093/eurheartj/ehv319. 26320109

[12] Cardoso T, Almeida M, Friedman ND, Aragão I, Costa-Pereira A, Sarmento AE, Azevedo L. 2014. Classification of healthcare-associated infection: a systematic review 10 years after the first proposal. BMC Med 12:40. doi:10.1186/1741-7015-12-40. 24597462 PMC4016612

[13] Schønheyder HC, Gottschau A, Friland A, Rosdahl VT. 1995. Mortality rate and magnitude of Staphylococcus aureus bacteremia as assessed by a semiquantitative blood culture system. Scand J Infect Dis 27:19–21. doi:10.3109/00365549509018967. 7784808

[14] Dahl A, Iversen K, Tonder N, Hoest N, Arpi M, Dalsgaard M, Chehri M, Soerensen LL, Fanoe S, Junge S, Hoest U, Valeur N, Lauridsen TK, Fosbol E, Hoi-Hansen T, Bruun NE. 2019. Prevalence of infective endocarditis in Enterococcus faecalis bacteremia. J Am Coll Cardiol 74:193–201. doi:10.1016/j.jacc.2019.04.059. 31296291

[15] Clinical and Laboratory Standards Institute (CLSI). 2022. Performance standards for antimicrobial susceptibility testing. 32nd ed. CLSI supplement M100. Pennsylvania Clinical and Laboratory Standards Institute, Wayne, PA.

[16] Facklam R. 2002. What happened to the streptococci: overview of taxonomic and nomenclature changes. Clin Microbiol Rev 15:613–630. doi:10.1128/CMR.15.4.613-630.2002. 12364372 PMC126867

[17] Roberts RB, Krieger AG, Schiller NL, Gross KC. 1979. Viridans streptococcal endocarditis: the role of various species, including pyridoxal-dependent streptococci. Rev Infect Dis 1:955–966. doi:10.1093/clinids/1.6.955. 551516

[18] Simmon KE, Hall L, Woods CW, Marco F, Miro JM, Cabell C, Hoen B, Marin M, Utili R, Giannitsioti E, Doco-Lecompte T, Bradley S, Mirrett S, Tambic A, Ryan S, Gordon D, Jones P, Korman T, Wray D, Reller LB, Tripodi MF, Plesiat P, Morris AJ, Lang S, Murdoch DR, Petti CA, International Collaboration on Endocarditis Microbiology Investigators. 2008. Phylogenetic analysis of viridans group streptococci causing endocarditis. J Clin Microbiol 46:3087–3090. doi:10.1128/JCM.00920-08. 18650347 PMC2546745

[19] Naveen Kumar V, van der Linden M, Menon T, Nitsche-Schmitz DP. 2014. Viridans and bovis group streptococci that cause infective endocarditis in two regions with contrasting epidemiology. Int J Med Microbiol 304:262–268. doi:10.1016/j.ijmm.2013.10.004. 24220665

[20] Netzer RO-M, Zollinger E, Seiler C, Cerny A. 2000. Infective endocarditis: clinical spectrum, presentation and outcome. An analysis of 212 cases 1980–1995. Heart 84:25–30. doi:10.1136/heart.84.1.25. 10862581 PMC1729423

[21] Sunder S, Grammatico-Guillon L, Lemaignen A, Lacasse M, Gaborit C, Boutoille D, Tattevin P, Denes E, Guimard T, Dupont M, Fauchier L, Bernard L. 2019. Incidence, characteristics, and mortality of infective endocarditis in France in 2011. PLoS One 14:e0223857. doi:10.1371/journal.pone.0223857. 31652280 PMC6814232

[22] Scheggi V, Merilli I, Marcucci R, Del Pace S, Olivotto I, Zoppetti N, Ceschia N, Andrei V, Alterini B, Stefàno PL, Marchionni N. 2021. Predictors of mortality and adverse events in patients with infective endocarditis: a retrospective real world study in a surgical centre. BMC Cardiovasc Disord 21:28. doi:10.1186/s12872-021-01853-6. 33435885 PMC7802147

[23] Suh Y, Kim M, Huh J, Cho O-H, Kim J-R, Kim S, Bae I-G. 2012. Factors associated with infective endocarditis and predictors of 3-month mortality of patients with viridans streptococcal bacteremia. Infect Chemother 44:419. doi:10.3947/ic.2012.44.6.419.

[24] Chamat-Hedemand S, Dahl A, Oestergaard L, Arpi M, Fosboel E, Boel J, Kaur KP, Oestergaard LB, Lauridsen TK, Gislason G, Torp-Pedersen C, Bruun NE. 2021. Independent risk factors of mortality in streptococcal infective endocarditis. European Heart J 42:ehab724.1713. doi:10.1093/eurheartj/ehab724.1713.

[25] Kang DH, Kim YJ, Kim SH, Sun BJ, Kim DH, Yun SC, Song JM, Choo SJ, Chung CH, Song JK, Lee JW, Sohn DW. 2012. Early surgery versus conventional treatment for infective endocarditis. N Engl J Med 366:2466–2473. doi:10.1056/NEJMoa1112843. 22738096

