# Supplementary material for: Risk and Outcome of Infective Endocarditis in Streptococcal Bloodstream Infections according to Streptococcal Species
Source: Microbiol Spectr. 2023 Jun 7;11(4):e01049-23. doi: 10.1128/spectrum.01049-23 (PMC10434186; doi:10.1128/spectrum.01049-23)
Supplement: Supplemental file 1 — Supplemental material. Download spectrum.01049-23-s0001.docx, DOCX file, 0.03 MB [file spectrum.01049-23-s0001.docx]

**Supplemental Table 1. Characteristics of patients with streptococcal bloodstream infections according to use of echocardiography (n = 2,737)**

| Characteristic | Pt. with Echo,  n = 1,177 (%) | Pt. without Echo,  n = 1,560 (%) | Total,  n = 2,737 (%) | *P* value |
| --- | --- | --- | --- | --- |
| **Age (yr), median (IQR)** | 62 (48–71) | 60 (48–69) | 61 (48–70) | 0.008 |
| **Male** | 688 (58.5) | 870 (55.8) | 1,558 (56.9) | 0.160 |
| **Community-onset BSI** | 923 (78.4) | 1,302 (83.5) | 2,225 (81.3) | <0.001 |
| **Underlying disease** |  |  |  |  |
| Congenital heart disease^a^ | 42 (3.6) | 9 (0.6) | 51 (1.9) | <0.001 |
| Uncorrected | 28 (2.4) | 6 (0.4) | 34 (1.2) | <0.001 |
| Corrected | 14 (1.2) | 3 (0.2) | 17 (0.6) | 0.001 |
| Native valve disease^b^ | 98 (8.3) | 41 (2.6) | 139 (5.1) | <0.001 |
| Prosthetic valve | 65 (5.5) | 4 (0.3) | 69 (2.5) | <0.001 |
| Cardiac device | 17 (1.4) | 1 (<0.1) | 18 (0.7) | <0.001 |
| Heart failure | 94 (8.0) | 23 (1.5) | 117 (4.3) | <0.001 |
| Ischemic heart disease | 73 (6.2) | 26 (1.7) | 99 (3.6) | <0.001 |
| **Streptococcal group** |  |  |  |  |
| Anginosus group | 296 (25.1) | 483 (31.0) | 779 (28.5) | <0.001 |
| Mitis group | 380 (32.3) | 367 (23.5) | 747 (27.3) | <0.001 |
| Pyogenic group | 245 (20.8) | 340 (21.8) | 585 (21.4) | 0.536 |
| Salivarius group | 97 (8.2) | 107 (6.9) | 204 (7.5) | 0.173 |
| Bovis group | 46 (3.9) | 41 (2.6) | 87 (3.2) | 0.059 |
| Mutans group | 16 (1.4) | 11 (0.7) | 27 (1.0) | 0.086 |
| Other streptococci^c^ | 111 (9.4) | 234 (15.0) | 345 (12.6) | <0.001 |
| **Streptococcal species** |  |  |  |  |
| *S. anginosus* | 208 (17.7) | 339 (21.7) | 547 (20.0) | 0.009 |
| *S. mitis* | 255 (21.7) | 258 (16.5) | 513 (18.7) | <0.001 |
| *S. agalactiae* | 159 (13.5) | 222 (14.2) | 381 (13.9) | 0.589 |
| *S. pneumoniae* | 111 (9.4) | 234 (15.0) | 345 (12.6) | <0.001 |
| *S. constellatus* | 73 (6.2) | 128 (8.2) | 201 (7.3) | 0.047 |
| *S. salivarius* | 94 (8.0) | 106 (6.8) | 200 (7.3) | 0.236 |
| *S. dysgalaciate* | 66 (5.6) | 79 (5.1) | 145 (5.3) | 0.530 |
| *S. oralis* | 67 (5.7) | 48 (3.1) | 115 (4.2) | <0.001 |
| *S. gallolyticus* | 44 (3.7) | 33 (2.1) | 77 (2.8) | 0.011 |
| *S. sanguinis* | 37 (3.1) | 27 (1.7) | 64 (2.3) | 0.015 |
| *S. pyogenes* | 21 (1.8) | 39 (2.5) | 60 (2.2) | 0.205 |
| *S. intermedius* | 15 (1.3) | 26 (1.7) | 41 (1.5) | 0.403 |
| *S. parasanguinis* | 15 (1.3) | 26 (1.7) | 41 (1.5) | 0.403 |
| *S. mutans* | 16 (1.4) | 11 (0.7) | 27 (1.0) | 0.086 |
| *S. gordonii* | 14 (1.2) | 8 (0.5) | 22 (0.8) | 0.050 |
| Other | 6 (0.5) | 9 (0.6) | 15 (0.5)^d^ | 0.814 |
| **High-grade BSI^e^** | 788 (66.9) | 889 (57.0) | 1,677 (61.3) | <0.001 |
| **Polymicrobial BSI** | 212 (18.0) | 393 (25.2) | 605 (22.1) | <0.001 |
| **Previous history of IE** | 15 (1.3) | 3 (0.2) | 18 (0.7) | <0.001 |

Data are numbers of patients (with corresponding percentages shown in parentheses) unless otherwise specified. Pt, patients; BSI, bloodstream infection; IE, infective endocarditis; IQR, interquartile range.

^a^Patients with both congenital heart disease and native valve disease or prosthetic valves were classified into only this group.

^b^Native valve disease includes aortic stenosis, aortic insufficiency, mitral stenosis, and mitral insufficiency.

^c^Other streptococci includes only *S. pneumoniae*.

^d^Of the 15 patients with other streptococci BSI, 6 had *S. lutetiensis* bacteremia, 4 *S. vestibularis*, 3 *S. equinus*, 1 *S. cristatus*, and 1 *S. alactolyticus*.

^e^High-grade bloodstream infection was defined as positive results obtained in over 50% of blood culture bottles with positive results for at least two bottles.

**Supplemental Table 2. Characteristics of patients with echocardiography according to infective endocarditis (n = 1,177)**

| Characteristic | Pt. with IE,  n = 174 (%) | Pt. without IE,  n = 1,003 (%) | *P* value |
| --- | --- | --- | --- |
| **Age (yr), median (IQR)** | 58 (44–69) | 62 (50–72) | 0.049 |
| **Male** | 97 (55.7) | 591 (55.9) | 0.433 |
| **Community-onset BSI** | 162 (93.1) | 761 (75.9) | <0.001 |
| **Underlying disease** |  |  |  |
| Congenital heart disease | 13 (7.5) | 29 (2.9) | 0.003 |
| Uncorrected | 6 (3.4) | 22 (2.2) | 0.316 |
| Corrected | 7 (4.0) | 7 (0.7) | 0.002 |
| Native valve disease | 41 (23.6) | 57 (5.7) | <0.001 |
| Prosthetic valve | 39 (22.4) | 26 (2.6) | <0.001 |
| Cardiac device | 4 (2.3) | 13 (1.3) | 0.300 |
| Heart failure | 19 (10.9) | 75 (7.5) | 0.122 |
| Ischemic heart disease | 10 (5.7%) | 63 (6.3) | 0.787 |
| Solid cancer | 21 (12.1) | 384 (38.3) | <0.001 |
| Diabetes mellitus | 28 (16.0) | 271 (27.0) | 0.002 |
| Liver cirrhosis | 15 (8.6) | 175 (17.3) | 0.003 |
| Chronic kidney disease | 12 (6.9) | 129 (12.9) | 0.003 |
| Chronic kidney disease on renal replacement | 4 (2.3) | 41 (4.1) | 0.400 |
| Hematologic malignancy | 2 (1.1) | 143 (14.3) | <0.001 |
| Chronic obstructive disease | 4 (2.3) | 33 (3.3) | 0.640 |
| **High-grade BSI** | 156 (89.7) | 632 (63.0) | <0.001 |
| **Polymicrobial BSI** | 10 (5.7) | 202 (20.1) | <0.001 |
| **Previous history if IE** | 12 (6.9) | 3 (0.3) | <0.001 |
| **Streptococcal group** |  |  |  |
| Mitis group | 79 (45.4) | 301 (30.0) | <0.001 |
| Anginosus group | 34 (19.5) | 262 (26.1) | 0.065 |
| Pyogenic group | 30 (17.2) | 215 (21.4) | 0.208 |
| Bovis group | 12 (6.9) | 34 (3.4) | 0.028 |
| Mutans group | 9 (5.2) | 7 (0.7) | <0.001 |
| Salivarius group | 6 (3.4) | 91 (9.1) | 0.013 |
| Other streptococci | 5 (2.9) | 106 (10.6) | 0.001 |
| **Streptococcal species** |  |  |  |
| *S. mitis* | 39 (22.4) | 216 (21.5) | 0.795 |
| *S. anginosus* | 29 (16.6) | 179 (17.8) | 0.706 |
| *S. agalactiae* | 22 (12.6) | 137 (13.7) | 0.718 |
| *S. pneumoniae* | 5 (2.9) | 106 (10.6) | 0.001 |
| *S. salivarius* | 6 (3.4) | 88 (8.8) | 0.017 |
| *S. constellatus* | 5 (2.9) | 68 (6.8) | 0.049 |
| *S. dysgalaciate* | 5 (2.9) | 61 (6.1) | 0.090 |
| *S. oralis* | 14 (8.0) | 53 (5.3) | 0.159 |
| *S. gallolyticus* | 12 (6.9) | 32 (3.2) | 0.017 |
| *S. sanguinis* | 20 (11.5) | 17 (1.7) | <0.001 |
| *S. pyogenes* | 3 (1.7) | 18 (1.8) | >0.99 |
| *S. mutans* | 9 (5.2) | 7 (0.7) | <0.001 |
| *S. gordonii* | 5 (2.9) | 9 (0.9) | 0.043 |
| *S. parasanguinis* | 1 (0.6) | 14 (1.4) | 0.712 |
| Other | 0 | 21 (2.1)^a^ | 0.059 |
| **Valve surgery within 3 months of BSI** | 93 (53.4) | 26 (2.6) | <0.001 |
| **Recurrence of BSI within 6 months** | 3 (1.7) | 7 (0.7) | 0.174 |
| **30-day mortality** | 10 (5.7) | 73 (7.3) | 0.466 |
| **90-day mortality** | 15 (8.6) | 145 (14.5) | 0.038 |
| **1-year mortality** | 40 (23.0) | 418 (41.7) | <0.001 |

Data are numbers of patients (with corresponding percentages shown in parentheses) unless otherwise specified. Pt, patients; BSI, bloodstream infection; IE, infective endocarditis; IQR, interquartile range.

^a^Of the 21 patients with other streptococci BSI, 15 had *S. intermedius*, 3 *S. vestibularis*, 2 *S. lutetiensis*, and 1 *S. cristatus* bacteremia.
