# Supplementary material for: Risk and Outcome of Infective Endocarditis in Streptococcal Bloodstream Infections according to Streptococcal Species
Source: Microbiol Spectr. 2023 Jun 7;11(4):e01049-23. doi: 10.1128/spectrum.01049-23 (PMC10434186; doi:10.1128/spectrum.01049-23)
Supplement: Supplemental file 2 — Supplemental material. Download spectrum.01049-23-s0002.docx, DOCX file, 0.6 MB [file spectrum.01049-23-s0002.docx]

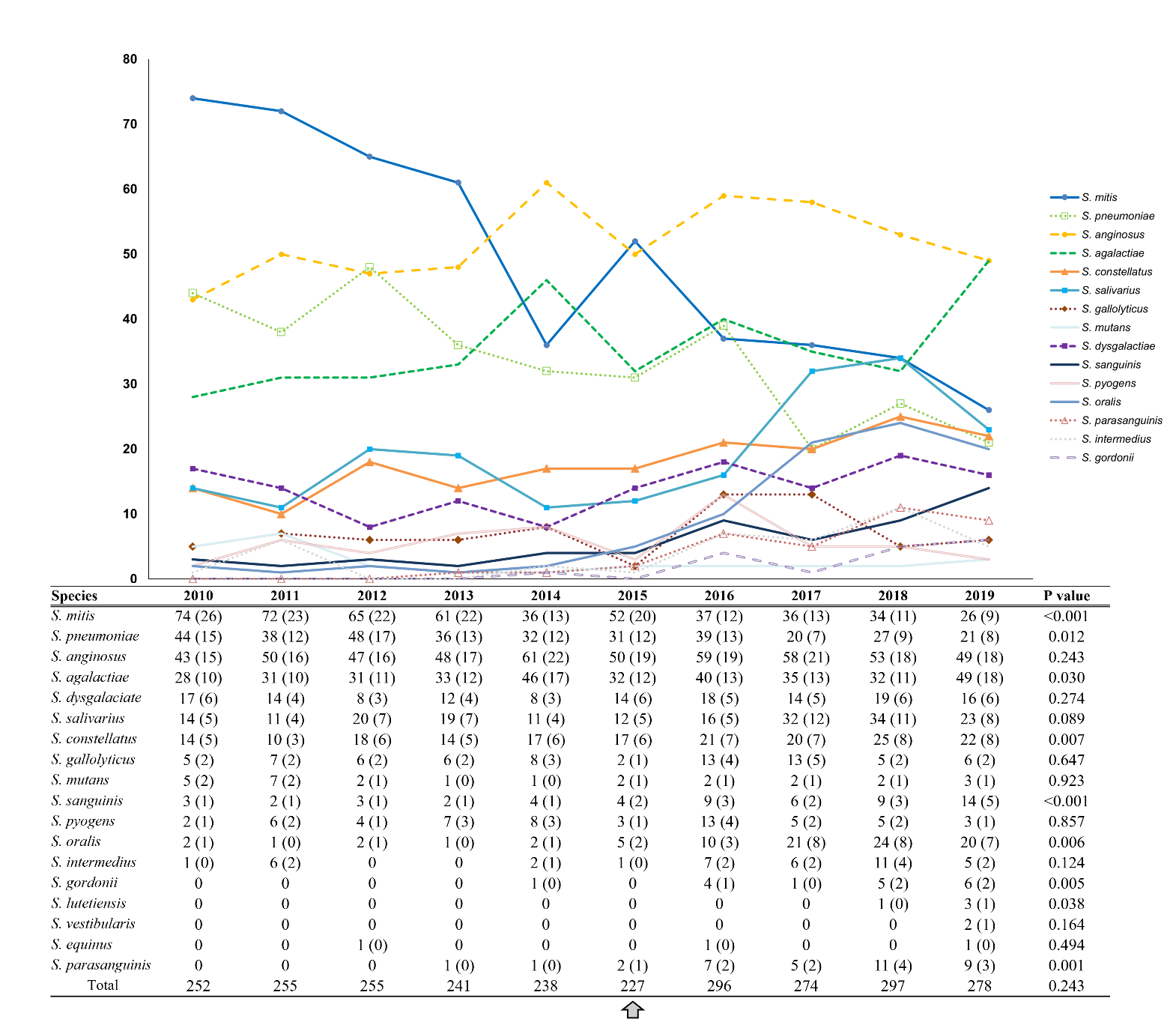


**Supplemental Figure 1. 10-year trends of streptococcal bloodstream infections.**

Data are presented as numbers of patients (with the corresponding percentages shown in parentheses). The vertical axis shows numbers of cases of bloodstream infections. Species with their total number above 10 are presented in the graph. The arrow indicates the year when MALDI-TOF was introduced in our center.
